# Unfolded Protein Corona Surrounding Nanotubes Influence the Innate and Adaptive Immune System

**DOI:** 10.1002/advs.202004979

**Published:** 2021-03-01

**Authors:** Jun‐Young Park, Sung Jean Park, Jun Young Park, Sang‐Hyun Kim, Song Kwon, YunJae Jung, Dongwoo Khang

**Affiliations:** ^1^ Lee Gil Ya Cancer and Diabetes Institute Gachon University Incheon 21999 South Korea; ^2^ Department of Health Sciences and Technology GAIHST Gachon University Incheon 21999 South Korea; ^3^ College of Pharmacy and Gachon Institute of Pharmaceutical Sciences Gachon University Incheon 21936 South Korea; ^4^ Department of Pharmacology School of Medicine Kyungpook National University Daegu 41944 South Korea; ^5^ Department of Microbiology School of Medicine Gachon University Incheon 21999 South Korea; ^6^ Department of Physiology School of Medicine Gachon University Incheon 21999 South Korea

**Keywords:** conformational changes, immune response, nanotubes, protein corona

## Abstract

The plasma proteins around nanoparticles (NPs) form an outer protein corona, significantly influencing the subsequent immune response. However, it was uncertain whether the protein corona around NPs influences immune response. This study clarified that the immune response mediated by the protein corona is greatly dependent on the type of plasma proteins surrounding the NPs. Structural changes in the unfolded protein corona elevated reactive oxygen species (ROS) levels and induced major proinflammatory cytokine release in both murine and human macrophage cell lines. In contrast, negligible structural changes in the protein corona provoke neither ROS production nor proinflammatory cytokine release. Furthermore, in vivo analysis confirms that a stimulated immune response by an unfolded protein corona triggers selective activation of innate and adaptive immunity in the spleen. Specifically, neutrophils, natural killer cells, and CD8^+^ T cells are overpopulated by unfolded protein corona structures surrounding nanotubes, whereas innate and adaptive immunologic responses are not triggered by a normal protein corona. In conclusion, highly unfolded protein corona structures are strongly correlated with subsequent activation of proinflammatory cytokines and innate immune responses; thus, the protein corona can be used in immune‐enhancing therapy.

## Graphical abstract

Schematic images illustrating structural changes in the plasma protein (unfolded) surrounding the nanotube that elicit innate and adaptive immune responses. Plasma proteins with a native structures surrounding nanotube did not induce immune responses.

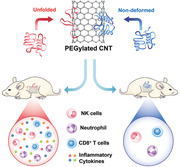

## 1 Introduction

The immunological impact of protein corona surrounding nanoparticles (NPs) has raised considerable concerns.^[^ ^1^ ^]^ Many plasma proteins allow NPs to interact with them, and the newly formed protein corona around an NP can influence the activation of the immune system.^[^ ^2^, ^3^ ^]^ The interaction between NPs and plasma proteins can induce structural changes highly dependent on the NP physicochemical properties.^[^ ^4^ ^]^ Importantly, the altered structure of plasma proteins can directly influence subsequent immune responses. In this regard, analyzing the structural changes in protein corona around the NPs and examining their associated influence on immune response is meaningful.^[^ ^5^, ^6^, ^7^ ^]^

Traditionally, “hard” corona (proteins bound with high affinity) has been considered to be more significant than “soft” corona (weak affinity for NP).^[^ ^8^, ^9^ ^]^ Thus, the immune cell predominantly sees the hard corona since a longer residence time increases the plasmatic uptake probability of immune cells.^[^ ^9^, ^10^ ^]^ In fact, it would not be very feasible to observe the immunological impact of a specific protein corona surrounding an NP since plasma protein corona around NPs is very transient and dynamically altered within a very short time.^[^ ^11^ ^]^ In this aspect, examining the immune response, triggered by a stable and constant type of specific proteins on NPs, is useful to understand the immunological influence of complex protein corona around NPs. Consequently, analyzing the conformational changes, depending on the type of plasma proteins induced by NPs, and elucidating their relationship with the immune system, can provide a more in‐depth insight into the mechanism of the differential response of human plasma proteins to NPs.

Previous study had analyzed the quantitative proteomics data of plasma protein corona interacting with NPs,^[^ ^12^ ^]^ and physiochemical properties (especially size) of NPs were found to be influential factors for determining a higher affinity with plasma proteins.^[^ ^13^ ^]^ In addition, complement activation was considered to be a “potential hazard” due to the altered protein corona.^[^ ^14^ ^]^ Deformation of fibrinogen (FN) adsorbed on gold NP triggered the downstream of NF‐*κ*B signal pathway and resulted in the release of proinflammatory cytokines.^[^ ^2^ ^]^ Therefore, the structural variation of plasma protein corona due to the interaction with NP was regarded as “another source” to activate subsequent inflammatory response and proinflammatory signal pathways, which were initiated by binding of specific plasma proteins on NP.^[^ ^2^, ^15^ ^]^ However, the mechanism underlying structural alteration of plasma protein in immunological response (innate or adaptive) in vivo is not fully elucidated. Immune responses in an in vivo model demonstrate how structural variations of protein corona can influence immune response at organized lymphoid tissues, such as lymph nodes and spleen.^[^ ^16^ ^]^

In this study, conjugation of *α*_1_ acid‐glycoprotein (AGP; acute phase plasma proteins in response to inflammation) and immunoglobulin G (IgG; the most common type of antibody secreted by B cells) with PEGylated carbon nanotubes (CNT) induced conformational changes in protein structures (unfolded). In contrast, FN (a glycoprotein that helps in the formation of blood clots) and vitronectin (VN; a glycoprotein that has been involved in tissue cell adhesion and proliferation), conjugated with CNT, do not induce any conformational rearrangements. As such, structural variation (i.e., unfolded protein corona surrounding CNT) of conjugated proteins depends on the type of attached proteins. Importantly, this study identified that the unfolded corona on CNTs significantly triggered an immune response both in vitro and in vivo. A non‐deformed protein corona, however, did not elicit an immune response.

## 2 Results

### 2.1 Structural Changes in Plasma Proteins Trigger Immune Response Activation

**Figure 1:**
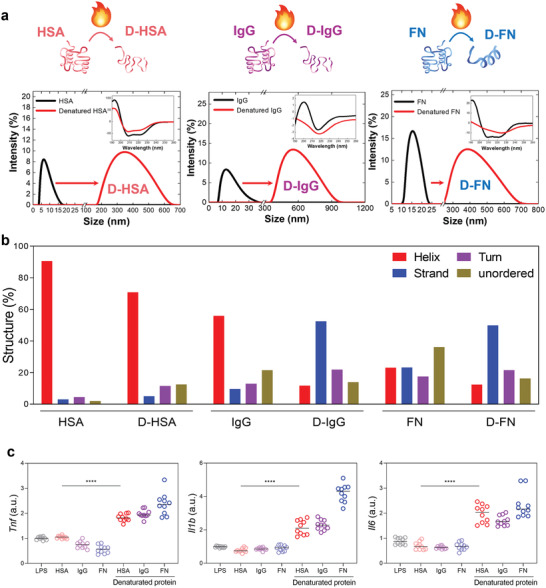
Denaturation of plasma proteins induce an inflammatory response. a) Schematic image shows heat‐induced protein denaturation with an increased size of proteins compared to nondenatured proteins. The inset image shows altered CD spectra of denatured protein (D‐HSA, D‐IgG, and D‐FN) compared to nondenatured proteins. Analysis of CD spectra clearly shows huge alteration in secondary structures by heat treatment. b) According to algorithm analysis, it was identified that denatured proteins have increased turn, strand, and unordered structures and a simultaneous decrease in helix structures (compared to nondenatured proteins). c) Significant increase in relative gene expression (*mRNA*) of proinflammatory cytokines (*Tnf, Il1b, Il6*) in J774A.1 after the treatment of denatured proteins. Nondenatured proteins show no upregulation of proinflammatory cytokines.

To examine the structural changes of plasma protein (unfolded protein) that can affect the activation of the immune response, serum albumin (HSA), and IgG and FN proteins were heated to deform the protein structures artificially. The proteins were boiled at a temperature of 90 °C for 30 min, which causes precipitation through denaturation of the secondary structures in the complexed protein (**Figure** **1**a and Figure S1a, Supporting Information). After cooling the precipitated (denatured by heat) protein solution for 30 min, only the supernatant of the protein solution was collected and used for further experiments. Denatured AGP highly aggregated in the aqueous state, and thus, HSA was used as a substitute model protein for further physiochemical analysis (Figure 1a). Due to denaturation, the hydrodynamic size of the denatured proteins was greatly increased with few micron meters from 5 to 20 nm of unfolded proteins (Figure 1a). Electric potential of each protein was found to be altered: −0.4 mV (denatured HSA) from 3.1 mV (HSA), +9.7 mV (denatured IgG) from −1.6 mV (IgG), and −10.7 mV (denatured FN) from −3.9 mV (FN) (Figure S1b, Supporting Information). The secondary structures of denatured plasma proteins were analyzed by far‐UV circular dichroism (CD) spectra (inset image of Figure 1a). Secondary structures of plasma proteins (HSA, IgG, and FN) were significantly influenced by the heat treatment. In short, the ratio of the helix structure was greatly reduced, whereas the ratio of the strand, turn, and unordered structures was significantly increased (Figure 1b).To investigate whether immunotoxicity is influenced by denatured proteins, the immune response was analyzed with the denatured proteins in murine macrophages (J774A.1). A significant increase in major proinflammatory cytokines such as tumor necrosis factor (*Tnf*), interleukin 1 beta (*Il1b*), and interleukin 6 (*Il6*) by denatured proteins in macrophages was observed; however, proinflammatory cytokines were not upregulated by nondeformed structures of plasma proteins (Figure 1c). The results obtained indicated that denatured proteins (with structural variation) induce the activation of major proinflammatory markers in innate immune responses (Figure 1c).

### 2.2 Physical Properties of Conjugated Protein Corona on Nanotubes

**Figure 2:**
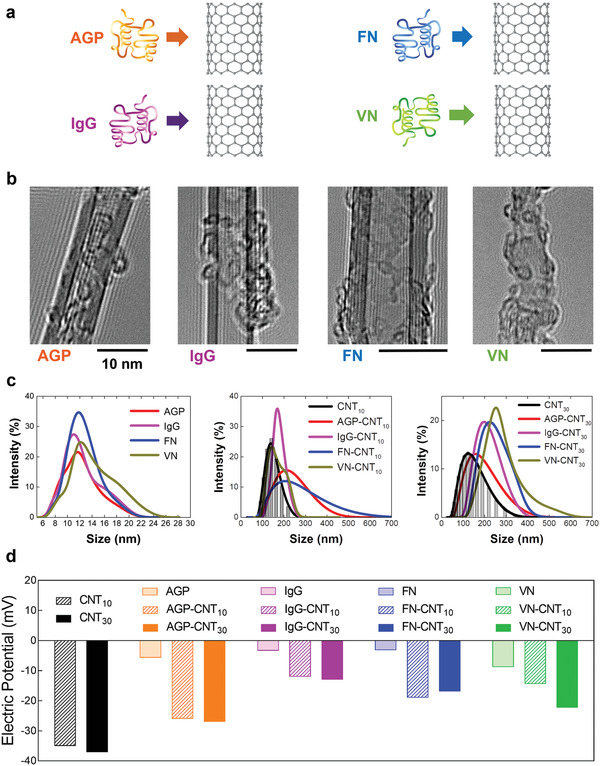
Physical characterization of protein corona around carbon nanotubes (CNTs). a) Schematic illustration of unfolded (AGP and IgG) and non‐unfolded (fibrinogen [FN] and vitronectin [VN]) protein corona–CNT conjugates. b) TEM images clearly show conjugated protein coronas around CNTs (scale bar is 10 nm). c) Hydrodynamic size of bare proteins (around 10 nm), various protein corona on CNT_10_, and protein corona on CNT_30_. Differences between the increased dimension of AGP, IgG, FN, and VN with CNTs were not significant. d) Zeta potential analysis shows that electric charges of various protein coronas on CNT_10_ and CNT_30_ shift toward negative charges compared to bare protein in PBS (pH 7.4).

As discussed in the introduction, hard corona surrounding NPs is more stable than soft corona.^[^ ^17^ ^]^ However, to obtain a consistent (in vitro and in vivo) immune response triggered by specific types of plasma proteins surrounding NPs, a more stable and stronger binding than the conventional hard corona on NPs would be beneficial. In this regard, an artificially formed plasma protein corona around CNTs, mimicking the hard corona, might help identify the resulting selective immune response. To this end, protein coronas (IgG, AGP, FN, and VN) were formulated by the conjugation of CNTs to mimic a stable “hard corona” surrounding the CNTs (**Figure** **2**a). Further, CNTs of two diameters (10 and 30 nm) were conjugated with plasma proteins to compare immune responses by the altered dimension of the interacting CNTs.

TEM images clearly showed a conjugated plasma protein corona around CNTs (Figure 2b). The hydrodynamic size of plasma proteins was approximately 10 nm (Figure 2c). After interaction with AGP, IgG, FN, and VN, the length of CNT_10_ (diameter of 10 nm) increased to 250 nm (AGP‐CNT_10_), 200 nm (IgG‐CNT_10_), 260 nm (FN‐CNT_10_), and 180 nm (VN‐CNT_10_) from 120 nm (average length of PEGylated CNT_10_), respectively (Figure 2c). The average length of CNT_30_ (diameter of 30 nm and length of 140 nm) after interaction with AGP, IgG, FN, and VN, increased to 150 nm (AGP‐CNT3_0_), 210 nm (IgG‐CNT_30_), 250 nm (FN‐CNT_30_), and 280 nm (VN‐CNT_30_), respectively (Figure 2c). The electric potential of each protein was found to be −5.6 mV (AGP), −3.3 mV (IgG), −3.2 mV (FN), and −8.8 mV (VN) at pH 7.2 (Figure 2d). All proteins (AGP, IgG, FN, and VN) had a weak negative charge due to their isoelectric point (PI); AGP had 2.7 to 3.5, IgG had 6.9, FN had 5.5, and VN had 5.67 (at pH > PI, proteins carry a net negative charge^[^ ^18^ ^]^). The electric potentials of CNT_10_ and CNT_30_ were −35 and −37 mV, respectively. Changes in electric potential after protein corona formation were slightly more positive than that of CNTs (Figure 2d). All conjugated proteins, AGP, IgG, FN, and VN on CNTs (both CNT_10_ and CNT_30_), show similar protein adsorption weight ratio (Figure S2, Supporting Information).

### 2.3 Conformational Change in Protein Corona is Selective Depending on the Types of Conjugated Proteins Around CNTs

**Figure 3:**
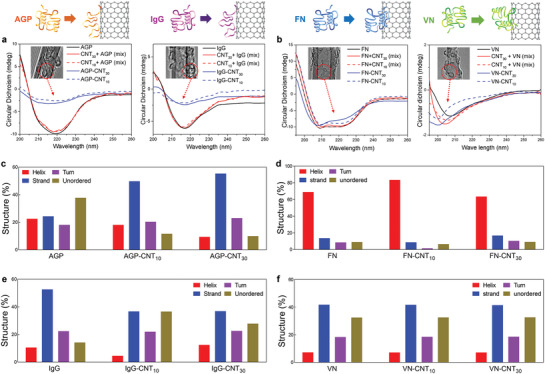
Structural changes of unfolded protein corona around carbon nanotubes (CNTs). a) The circular dichroism (CD) analysis shows that the secondary structures of the unfolded protein corona (AGP‐CNTs and IgG‐CNTs) are significantly influenced by the conjugation with CNTs. b) In contrast, a structural variation of FN‐CNTs and VN‐CNTs were insignificant compared with FN and VN, respectively. c) Relative changes in the secondary structure of AGP were significantly influenced by their interaction with CNTs (both CNT_10_ and CNT_30_). d) Changes in the secondary structure of FN were not affected at all by interaction with CNTs (both CNT_10_ and CNT_30_). e) Relative changes in the secondary structure of IgG were significantly influenced by their interaction with CNTs (both CNT_10_ and CNT_30_). f) Changes in VN secondary structure were not influenced by the interaction with CNTs (both CNT_10_ and CNT_30_). The solutions containing plasma proteins and CNTs as individual entities are denoted with “mix,” and the solutions of protein‐CNT‐conjugated complexes are depicted as “protein‐CNT.”

To investigate conformational changes of conjugated proteins surrounding CNTs, the secondary structures of the plasma protein corona were analyzed by far‐UV CD spectra (**Figure** **3**a–f and Figure S3, Supporting Information). After conjugation with CNTs, secondary structures of AGP and IgG were significantly influenced, whereas simple mixing of CNTs with AGP and IgG did not cause any notable change in the protein structure (Figure 3a,c,e). Specifically, the secondary structure of AGP and IgG was significantly altered, and the extent of change of the secondary structure was further increased in CNTs with a smaller diameter (10 nm) than in those with a larger diameter (30 nm) (Figure 3a,c). Observed results coincide with previous ones, suggesting that smaller diameter CNTs induce greater conformational changes.^[^ ^19^, ^20^ ^]^ In contrast, FN and VN exhibited a negligible difference of conformational change in the secondary structure, regardless of the interacting CNT size (Figure 3d,f). The secondary structure of all tested proteins was further analyzed by the CDSSR method.^[^ ^21^ ^]^ Specifically, the secondary structure of AGP was originally composed of 57.08% of *α*‐helices, 18.52% of *β*‐strands, 13.71% of turns, and 10.71% of unordered structures. After conjugation with CNTs, the AGP *α*‐helical structure most significantly decreased from 57.06% to 23.02% with CNT_30_ and from 57.06% to 21.39% with CNT_10_. In the case of IgG, *α*‐helix decreased from 35.19% to 6.63% in the presence of CNT_30_ and from 35.91% to 4.6% with CNT_10_ (Figure 3c,e). In short, the relative changes in the *α*‐helix structure of AGP and IgG were significantly influenced by their interaction with CNTs (both CNT_10_ and CNT_30_), and unordered/turn structures replaced the accompanying decrease of *α*‐helix structures (Figure 3c,e). In contrast, there was no apparent structural change in FN and VN (Figure 3d,f), regardless of the size of the CNTs (both 10 and 30 nm). Thus, for the first time, our results supported the evidence that change in the secondary structure of corona (unfolding corona) on CNTs is dependent on the type of conjugated plasma proteins.

### 2.4 Release of Proinflammatory Cytokines by Unfolded Protein Corona Around CNT

**Figure 4:**
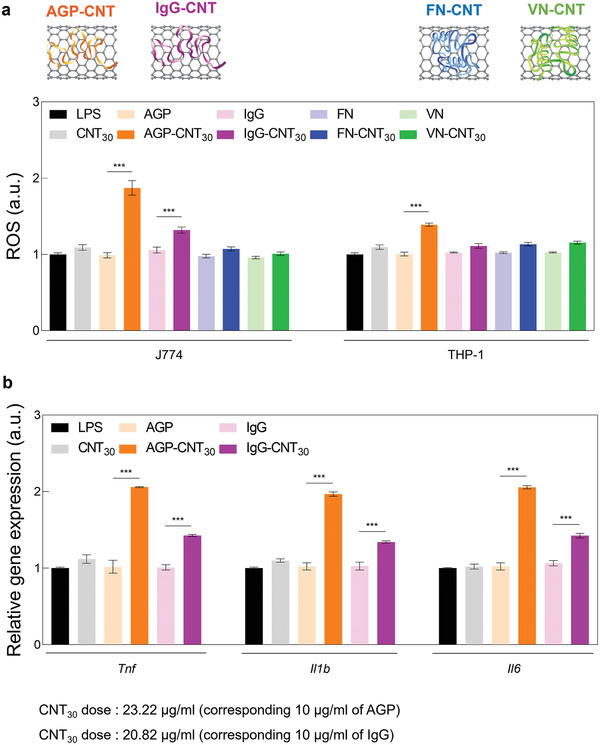
Reactive oxygen species (ROS) and proinflammatory cytokines by unfolded corona around carbon nanotubes (CNT). a) ROS levels in J774A.1 and THP‐1 treated with unfolded corona groups (AGP‐CNT_30_ and IgG‐CNT_30_) were significantly increased compared to those of normally structured corona groups (FN‐CNT_30_ and VN‐CNT_30_). b) mRNA level of *Tnf*, *Il1b*, and *Il6* in J774A.1 treated with unfolded protein corona (AGP‐CNT_30_ and IgG‐CNT_30_) significantly increased compared to other groups.

Previous studies from other groups have shown that NPs play a significant role in determining conformational changes of corona proteins and subsequent cellular interactions.^[^ ^20^, ^22^ ^]^ However, studies of immune response associated with conformational changes in the protein corona surrounding NPs have been rare and not comprehensively understood yet.^[^ ^2^, ^23^ ^]^ To examine how immunotoxicity is influenced by the conformational changes in protein corona, examining reactive oxygen species (ROS) production by macrophages and monocytes after treatment of unfolded corona and normally structured corona is a prerequisite before subsequent proinflammatory cytokine analysis.^[^ ^5^ ^]^ J774A.1 (murine macrophage) and THP‐1 (human monocyte) cells were incubated with unfolded and normal structures of proteins on CNT_30_ at identical concentrations (IgG, AGP, FN, and VN) for 2 h (after 12‐h of Lipopolysaccharides (LPS) stimulation), and ROS production was measured by 2′, 7′‐dichlorodihydrofluorescein diacetate (DCF‐DA) (**Figure** **4**a). Results showed that both macrophages and monocytes induced the production of inflammatory mediator ROS.^[^ ^24^ ^]^ In particular, an unfolded corona (AGP‐CNT_30_ and IgG‐CNT_30_) resulted in significant ROS production, whereas normal coronas (FN‐CNT_30_ and VN‐CNT_30_) did not trigger any notable ROS generation in LPS‐treated macrophages and monocytes (Figure 4a). Furthermore, the smaller diameter CNT_10_ induced more ROS than the larger diameter CNT_30_ and also increased the release of proinflammatory cytokine compared to the larger diameter CNTs (Figure S4, Supporting Information). Interestingly, normal structured proteins surrounding CNT_30_ (FN‐CNT_30_ and VN‐CNT_30_) did not induce any notable changes in ROS production in macrophages and monocytes (Figure 4a).

Furthermore, unfolded corona structures on CNTs (AGP‐CNT_30_ and IgG‐CNT_30_) induced significant release of major proinflammatory cytokines, such as *Tnf*, *Il1b*, and *Il6* in macrophages (J774A.1). In contrast, such proinflammatory cytokine expression was not induced by the normally structured corona surrounding CNT_30_ (FN‐CNT_30_ and VN‐CNT_30_) (Figure 4b). The smaller diameter of CNT_10_ induced more proinflammatory cytokines than the larger diameter of CNT_30_ (Figure S4, Supporting Information). Therefore, the structural changes of unfolded plasma protein corona surrounding CNTs significantly influenced subsequent immunotoxicity due to the induction of ROS and proinflammatory cytokines. The standard structured protein corona on CNTs (FN‐CNT_10_ and VN‐CNT_30_), however, resulted in negligible ROS (Figure 4a). Our observations collectively indicate that the structural changes of unfolded plasma protein corona surrounding CNTs promote the production of inflammatory mediators of early inflammatory, including ROS and proinflammatory cytokines.

### 2.5 Comparison of in vivo Proinflammatory Cytokine Analysis by Unfolded Proteins Corona Around CNTs

**Figure 5:**
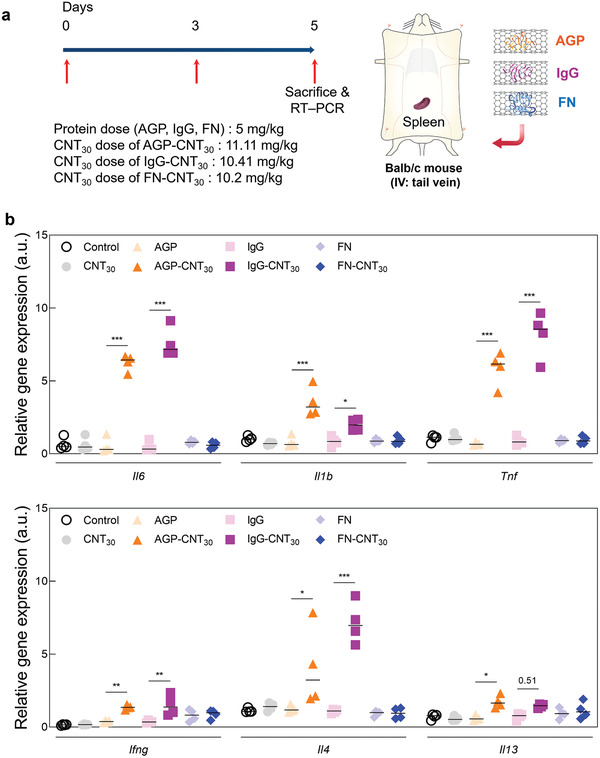
Immune response by unfolded corona protein around carbon nanotubes (CNTs) (spleen): Short‐term analysis. a) The schematic illustration represents IV injection of unfolded protein corona to examine the short‐term immune response of the spleen. (b) Relative gene expression (*mRNA*) of innate inflammatory cytokines (*Il6, Il1b, Tnf, Ifng, Il4, and Il13*) in the spleen after short‐term injections of unfolded protein corona surrounding CNT_30_. The unfolded protein corona structures (AGP‐CNT_30_ and IgG‐CNT_30_) significantly increased inflammatory cytokines in the spleen compared to FN‐CNT_30_.

**Figure 6:**
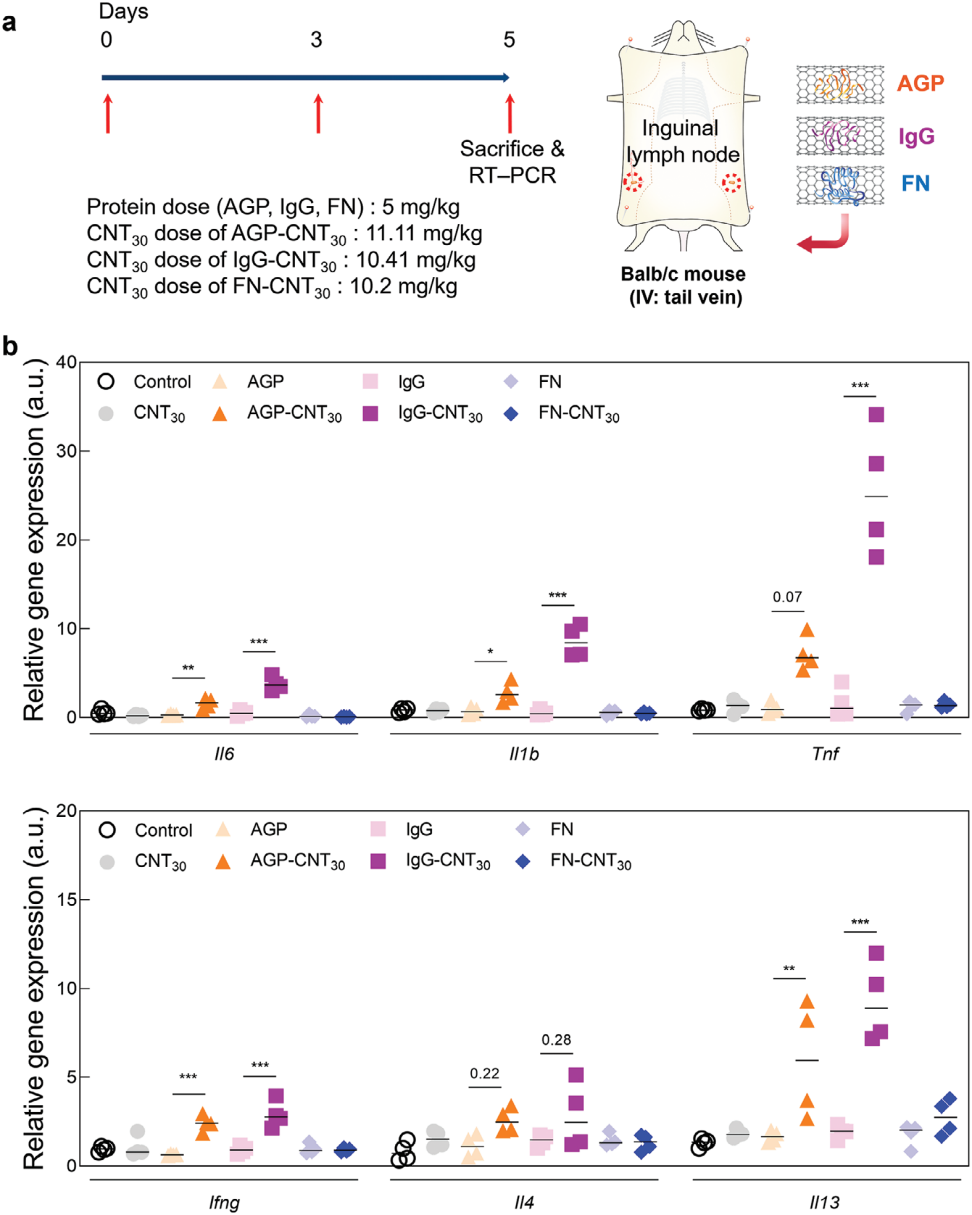
Immune response by unfolded corona protein around carbon nanotubes (CNTs) (lymph node): Short‐term analysis. a) The schematic illustration represents i.v. injection of unfolded protein corona to examine the short‐term immune response of the inguinal lymph nodes. b) Relative gene expression (*mRNA*) of innate inflammatory cytokines (*Il6, Il1b, Tnf, Ifng, Il4, and Il13*) in inguinal lymph nodes after short‐term injections of unfolded protein corona surrounding CNT_30_. The unfolded protein corona structures (AGP‐CNT_30_ and IgG‐CNT_30_) significantly increased inflammatory cytokines in the spleen compared to FN‐CNT_30_.

The biodistribution analysis shows that protein coronas surrounding CNTs (CNT with a diameter of 30) accumulated in spleens and lymph nodes after 24 h of post‐injection (Figure S5, Supporting Information). Considering various innate and adaptive immune cells located in the spleen and lymph nodes (inguinal lymph nodes), immunological interactions with protein corona are unavoidable. Immune responses in the spleen and lymph node are highly susceptible to inflammatory triggers, as the spleen is highly vascularized and lymph nodes are located along lymphatic channels throughout the body.^[^ ^25^ ^]^ To examine the inflammatory responses provoked in the spleen and lymph node by the corona surrounding the CNTs, unfolded or nondeformed proteins were intravenously injected in mice twice every 3 days (5 mg kg^−1^ of protein dose. **Figures** **5**a and **6**a). However, the administration of CNTs and individual proteins (AGP and IgG) in the spleen did not result in any notable expression of proinflammatory cytokines in the spleen, whereas the mRNA expression of innate (*Il6, Il1b*, and *Tnf*) and adaptive (interferon gamma (*Ifng*) and interleukin 4 (*Il4*)) cytokines were significantly upregulated by the unfolded corona proteins surrounding the CNTs (Figure 5b). In the lymph node, the expression of Il6, Il1b, Ifng, and interleukin13 (*Il13*) was significantly upregulated by the unfolded protein corona surrounding CNT_30_ (AGP‐CNT_30_ and IgG‐CNT_30_) (Figure 6b). However, the normal structured protein, FN‐CNT_30_ has not induced any inflammatory mediators in the spleen and lymph nodes (Figures 5b and 6b). Identical in vivo results were obtained for smaller diameter of CNTs (CNT_10_: see Figure S6, Supporting Information). Compared to in vitro analysis, in vivo analysis shows that upregulation of cytokines by the smaller diameter of CNT (CNT_10_) is not an influential factor for generating different innate and adaptive immune responses compared to the larger diameter of CNT (CNT_30_) statistically (Figure S7, Supporting Information).

### 2.6 Innate and Adaptive Immune Cell Response by Unfolded Protein Corona Around CNTs

**Figure 7:**
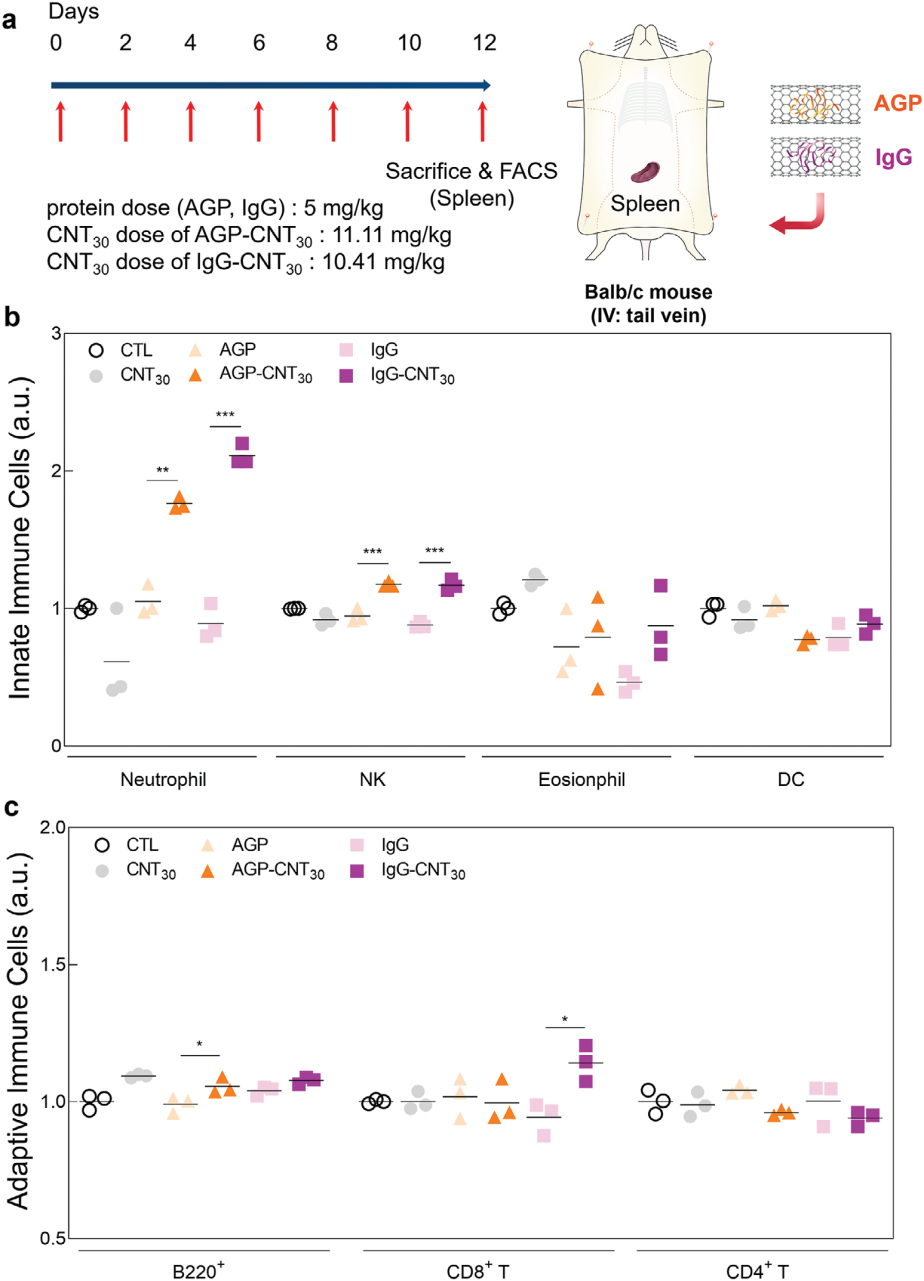
Immune response by unfolded corona protein around carbon nanotubes (CNTs) (spleen): Long‐term analysis. a) The schematic illustration represents IV injection of unfolded protein corona to examine the long‐term immune response of the inguinal lymph nodes. b) FACS analysis of isolated innate immune cells (neutrophil, NK, eosinophil, and DC) from spleen tissues showed a selective increase of neutrophil and NK cell population. c) FACS analysis of B and T cells (CD8^+^ and CD4^+^) isolated from the spleen exhibited a slight increase in CD8+ T cell population.

Next, authors hypothesize that unfolded protein corona surrounding CNTs might promote specific immune cell proliferation in the lymphoid organ. Authors examined the immune cell population in the spleen injected with unfolded and nondeformed protein to explore this idea. Based on a short‐term analysis (two injections every 3 days), significant changes in innate (neutrophil, eosinophil, and macrophage) and adaptive (B, CD4^+^ T and CD8^+^ T cells) immune cell frequency in mice due to the unfolded protein corona surrounding CNT_30_ were not observed (Figure S7, Supporting Information). Although the peak levels in the synthesis of immune mediators can be achieved within a few hours upon inflammatory triggers, differentiation and active proliferation of immune cells require several days after stimulation.^[^ ^26^ ^]^ Therefore, unfolded protein corona (both AGP‐CNT_30_ and IgG‐CNT_30_) were administrated six times for 10 days (every 2 days) (**Figure** **7**a). Interestingly, the repetitive and prolonged injection of AGP‐CNT_30_ and IgG‐CNT_30_ induced a significant increase in neutrophils, natural killer cell (NK cell), and CD8^+^ T cell levels in the spleen of mice compared with groups treated only with AGP or IgG (Figure 7b,c). The frequency of eosinophils and dendritic cells was unaffected by unfolded AGP and IgG surrounded CNT_30_ (Figure 7b). Considering that CD8^+^ T cells are more sensitive than CD4^+^ T cells in response to proliferation‐inducing antigenic signals,^[^ ^27^ ^]^ it is plausible that unfolded protein corona surrounding CNTs induces T‐cell activation in a similar way to antigen and costimulatory signals. It is worth to mention that population of effector T helper cells (CD4^+^) was increased whereas, population of naïve CD4^+^ T cells was decreased by unfolded protein corona (as such, total CD4^+^ cell population was identical) (Figure S8, Supporting Information).

Based on all obtained results, it is anticipated that short‐term (two injections) administration of unfolded protein corona (both AGP‐CNT_30_ and IgG‐CNT_30_) was insufficient to induce immune cell populations. However, it seems that repetitive administration of CNTs surrounded by unfolded protein corona was necessary to trigger the proliferation of activated immune cells. Therefore, it is speculated that the repetitive administration of unfolded protein corona on surrounding CNTs (less than 2 weeks) can significantly influence subsequent innate immune response and T‐cell responses. As such, altered protein corona structures are a critical factor for activating innate immune response and can initiate further adaptive immune responses. Long‐term administrations (more than several weeks) of unfolded protein corona structures in mice can clarify whether the adaptive immune response is sufficiently triggered by an unfolded protein corona. Nevertheless, further analysis is warranted to address this question.

### 2.7 Toxicological Evaluation

**Figure 8:**
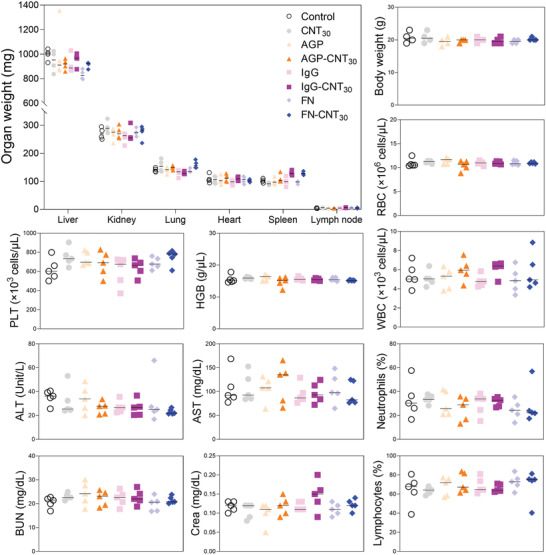
In vivo toxicity by unfolded corona proteins around carbon nanotubes (CNT). a) The weights of major organs after the treatments of saline, CNT, proteins (AGP, IgG, and FN), unfolded protein corona (AGP‐CNT_30_ and IgG‐ CNT_30_), and non‐unfolded protein corona (FN‐CNT_30_) shows no notable liver and Kinney toxicity between tested groups. All experimental groups were analyzed after collecting the samples from anesthetized murine models.

The preclinical in vivo analysis showed no significant changes in body and organ weights (i.e., liver, kidney, lung, heart, spleen, and lymph nodes) after six injections of the unfolded protein corona (**Figure** **8**). Besides, negligible liver toxicities were observed between all tested groups by analyzing alanine aminotransferase (ALT) and aspartate aminotransferase (AST) levels (Figure 8). Similarly, after analyzing the changes in phosphorus creatinine (Crea) and blood urea nitrogen (BUN), kidney toxicities did not significantly differ between groups. Finally, peripheral circulating white blood cell counts (i.e., leukocytes (WBC), lymphocytes, and neutrophils) were unchanged between groups (Figure 8).

## 3 Conclusion

The interaction of plasma proteins surrounding NPs with immune cells is an intriguing immunological issue. Based on previous studies, it is evident that altered protein structures on NPs can influence immune‐active functions of immune cells.^[^ ^7^ ^]^ Some previous studies also suggested a possible relationship between structural variation and immune response in vitro.^[^ ^28^ ^]^ However, a more detailed classification that elucidates how protein corona can influence immune cells is currently lacking. Notably, examining one or two plasma proteins without significant conformational changes is insufficient for identifying the impact of protein corona on the immune system. Second, examining the immune activity of both spleen and lymph nodes as sources of immune systems is essential to understand how the immune system responds to altered protein corona structures surrounding NPs.

In this study, several novel findings have been discussed. First, the structural variation of plasma proteins is selective; some plasma proteins are easily unfolded by the conjugation of CNTs, whereas the structural deformation of other proteins is negligible. Second, more remarkable changes in protein structures induce subsequent inflammatory responses both in vitro and in vivo. In contrast, the negligible structural alteration of plasma proteins did not result in any positive immunity either in vitro or in vivo.

Immune‐triggering proteins are highly sensitive to interaction with NP, and thus, immune systems can directly respond to unfolded plasma structures. Indeed, our natural defense system is designed to highly respond to injected NPs. Since reticuloendothelial system is the primary source of NP accumulation, further safety guidelines for the clinical use of NPs require an immune‐toxic response of accumulated plasma protein surrounding NP by examining major immune‐responsive organs, such as spleen and lymph nodes. In another aspect, however, an immune activation strategy using unfolded immune‐triggering plasma proteins around NPs can apply to the upregulation of immune activity at the tumor micro‐environment to increase the immune activity, thereby destroying tumor cells. With the injection of AGP‐CNT_30_ and IgG‐CNT_30_, a significant increase of NK cells in innate immunity and CD8+ T cells in adaptive immunity was observed. Although NK cells and CD8^+^ T cells have different target recognition mechanisms and signaling activations, both cells can be specialized to use cancer immunotherapy to increase immune‐toxic activity to cancer cells.^[^ ^29^ ^]^ Therefore, unfolded plasma proteins around NPs could be used for upregulating the levels of immune cells in the tumor microenvironment; thus, the suggested novel strategy can be used along with conventional immunotherapy. The information obtained from this study covers the immunological aspect and suggests a novel strategy for curing other diseases with the help of controlled immune systems, made possible by altered structures of unfolded protein corona surrounding NPs.

## 4 Experimental Section

##### Materials Preparation

Purified CNTs, with an outer diameter of <10 nm (CNT_10_, 900–1255, SES, USA), and 30 nm, were obtained (CNT_30_, 900–1260, SES, USA) and oxidized following a previously described protocol.^[^ ^30^ ^]^ Amino‐propyl PEG (PEG, MW = 5 kDa, NOF Corp., Japan) was used for generating PEG‐coated CNTs. Briefly, purified CNTs were preheated at 300 °C for 3 h to fully discard any vapor or contaminants, and sulfuric acid and nitric acid were subsequently added to the preheated CNT powder in a ratio of 3:1. The solution was sonicated for 99 min using a water bath sonicator and stirred for 36 h at 50 °C to generate a carboxyl group. After the stirring for 36 h, the CNT solution was diluted with distilled (DI) water (1:200 v/v) and filtered with a membrane filter (200 nm, PTFE, Millipore). The collected CNTs were dried in a vacuum oven at 60 °C for further use. The dried CNTs were washed with DI water three times to remove acidic residues through centrifugation at 4000 rpm for 10 min. The carboxylic acid‐functionalized CNT solution after adding amino‐propyl PEG (PEG, MW = 5 kDa, NOF Corp., Japan) was sonicated for 20 min in ice and then allowed to cool for 10 min. This process was repeated at least three times and washed with 2‐morpholinoethanesulfonic acid (MES) buffer (50 × 10^−3^ m, pH 8.0, M3671, Sigma) to remove unbound PEG. AGP, IgG, FN, and VN were obtained from Sigma‐Aldrich (G9885, I4506, F4883, A9511, and 5051, respectively). Proteins were noncovalently bonded but PEGylated to the CNTs through various interactions (i.e., *π*−*π*, polar−*π*, electrostatic interaction, and Van der Waals forces).^[^ ^31^ ^]^ Plasma protein‐CNT (i.e., AGP‐CNT, IgG‐CNT, FN‐CNT, and VN‐CNT) conjugation was used to investigate the immunotoxicity based on the extent of conformational changes in the protein corona depending on the type of plasma protein surrounding the CNTs.

##### Protein Conjugation and Physical Characterization

The PEGylated CNT solution was washed with MES buffer (50 × 10^−3^ m, pH 8.0, M3671, Sigma) at least thrice before conjugating with proteins. Selected plasma proteins (i.e., AGP, IgG, FN, and VN) were prepared at a concentration of 1 mg mL^−1^ in the same MES buffer and added to PEGylated CNT solution for conjugation. The mixtures were incubated for 48 h at 4 °C with shaking. The immune corona on PEGylated CNTs (i.e., AGP‐CNT, IgG‐CNT, FN‐CNT, and VN‐CNT) in MES buffer was filtered (Amicon YM‐50, 100 kDa, Millipore) at least thrice, followed by centrifugation at 4000 rpm to remove unconjugated proteins. After the conjugation process was over, plasma protein corona‐CNT (i.e., AGP‐CNT, IgG‐CNT, FN‐CNT, and VN‐CNT) complexes were maintained in PBS (pH 7.2) until physical characterization. After drying the sample in a vacuum oven at 60 °C for at least 2 days, the percentage of conjugated proteins on CNTs was calculated by measuring the weight variation between completely dried CNTs and dried plasma protein‐CNTs on a PTFE membrane (JGWP04700, Omnipore). The diameter and zeta potential of the complex in PBS (pH 7.2) were measured by dynamic light scattering and electrophoretic light scattering (Zetasizer Nano, Malvern, UK).

##### Circular Dichroism Spectroscopy

CD spectra were analyzed by a Chirascan CD spectrometer (Applied Photophysics, Randalls, UK). Data were collected at room temperature in the wavelength range of 190–260 nm, using a 1‐nm quartz cuvette (300 µL). Samples were prepared at 0.2 mg mL^−1^ concentration in sodium phosphate buffer (pH 7.4, 25 × 10^−3^ m). Spectral data were acquired at a 1‐nm bandwidth, 0.5 s per point, and each spectrum was obtained with five accumulations (averaged). Secondary structure content from the CD spectra was analyzed by CDPro software using the CDSSTR algorithm.

##### Cell Culture

The BALB/c macrophage cell line J774A.1 was obtained from the American Type Culture Collection (ATCC, TIB‐67, Manassas, VA, USA). Cells were cultured in Dulbecco's modified Eagle medium (Gibco, 11995‐065, Waltham, MA, USA) and supplemented with heat‐inactivated 10% fetal bovine serum (FBS, Gibco, 16000–044) and 1% penicillin/streptomycin (Gibco, 10 378 016) at 37 °C in 5% CO_2_). The human monocytic cell line THP‐1 was obtained from ATCC (TIB‐202, Manassas, VA, USA). Cells were cultured in RPMI 1640 medium (Gibco, 11875‐093) and supplemented with non‐heat inactivated 10% FBS (Gibco, 16000–044) and 1% penicillin/streptomycin (Gibco, 10378016) at 37 °C in 5% CO_2_. Cells (5 × 10^3^ cells well^−1^ in 96‐well plates) were treated with various concentrations of plasma proteins (i.e., IgG, AGP, FN, and VN), PEGylated CNTs, and plasma protein‐CNTs (i.e., IgG‐CNT, AGP‐CNT, FN‐CNT, and VN‐CNT) for 24 h.

##### Detection of Intracellular ROS

ROS was measured by detecting the fluorescence intensity of oxidant‐sensitive probes. J774A.1 and THP‐1 cells (5 × 10^3^ cells well^−1^ in 96‐well plates) were treated with various concentrations of plasma proteins (i.e., IgG, AGP, FNG, and VN), PEGylated CNTs, and plasma protein‐CNTs (i.e., IgG‐CNT, AGP‐CNT, FN‐CNT, and VN‐CNT) for 2 h, followed by stimulation with LPS (50 ng mL^−1^, L5293, Sigma) for 12 h. Cells were added to a PBS solution containing 10 × 10^−6^ m 2′, 7′‐dichlorodihydrofluorescein diacetate (DCF‐DA, D6883, Sigma), and intracellular and extracellular dye concentrations were allowed to equilibrate at 5% CO_2_ at 37 °C for 30 min. A change in the fluorescence intensity of the DCF‐DA was recorded by a Victor X3 multi‐label plate reader (Perkin Elmer, USA) at an excitation of 480 nm and emission of 525 nm.

##### Real‐Time Polymerase Chain Reaction (RT‐PCR)

To measure cytokine expression, quantitative real‐time PCR (CFX 96, Bio‐Rad, Hercules, CA, USA) was used in accordance with the manufacturer's protocol. J774A.1 was pretreated with immune‐triggering proteins (IgG and AGP), PEGylated CNTs, and immune trigging protein‐CNT complexes (IgG‐CNT and AGP‐CNT) for 2 h, followed by stimulation with LPS (50 ng mL^−1^) for 12 h. Total RNA was isolated from cells (5 × 10^5^ cells well^−1^ in 6‐well plates) and spleen using QIAzol lysis reagent (#79 306, Qiagen, Hilden, Germany) and subsequently purified with an RNeasy Mini Kit (#74 104, Qiagen). RNA (500 ng) was treated with DNase I (New England Biolabs, Ipswich, MA, USA) and cDNA was synthesized using an iScript cDNA Synthesis Kit (Bio‐Rad, Hercules, CA, USA). Reverse transcription conditions included 45 °C for 60 min and 95 °C for 5 min. Briefly, 2 µL of cDNA (500 ng mL^−1^), 1 µL of sense and antisense primer solutions (0.4 × 10^−6^ m), 12.5 µL of iQ SYBR Green supermix (170‐8880, Bio‐Rad), and 9.5 µL of dH_2_O were mixed to obtain a final 25‐µL reaction mixture in each reaction tube. PCR was carried out with the primer sequences listed in Table S1, Supporting Information (in vitro) and Table S2, Supporting Information (in vivo). Cycle threshold (*C*_t_) values were calculated using a CFX96 Real‐Time PCR Detection System (Bio‐Rad) software, and the comparative *C*_t_ method (2−Δ*C*_t_ model) was used to calculate relative fold‐changes in gene expression, which were subsequently normalized to the averaged GAPDH expression.

##### Animal Test

BALB/c mice (female, 4–6 weeks old, and 16–20 g) were purchased from Orient Bio (Seoul, Korea). After shipment, the mice were housed in a temperature‐ and humidity‐controlled, specific pathogen free environment with a 12‐h light/dark cycle (lights on at 6:30 AM) for 7–14 days, and allowed to acclimatize before the experiment. All animal experiments were carried out in accordance with the Guide for the Care and Use of Laboratory Animals of Gachon University. CNT‐conjugated denatured proteins (i.e., IgG‐CNT and AGP‐CNT), non‐denatured protein (FN‐CNT), and free proteins (i.e., IgG, AGP, and FN) were injected into the female BALB/c mice by intravenous injection at a protein concentration of 5 mg kg^−1^ in saline solution. CNT‐conjugated plasma proteins and unconjugated plasma proteins were injected twice every 3 days for a week. All animals were sacrificed 2 days after the end of protein injection.

##### Flow Cytometry Analysis of Innate and Adaptive Immune Cells

To characterize the surface phenotype of splenocytes, spleen cells were isolated as described previously.^[^ ^32^ ^]^ In brief, the spleen was gently disrupted, filtered through a nylon mesh to remove any aggregate, and treated with ammonium chloride‐potassium lysis buffer to lyse erythrocytes. Isolated cells were re‐suspended in FACS buffer (PBS containing 10% FCS, 20 × 10^−3^ m HEPES, and 10 × 10^−3^ m EDTA). After blocking Fc receptor with anti‐mouse CD16/CD32 (2.4G2, BD Biosciences, San Diego, CA, USA) for 15 min at 4 °C, each cell type was stained for 30 min at 4 °C with the following antibodies. Neutrophils were stained with mAbs against CD11b and Gr‐1 purchased from BioLegend (San Diego, CA, USA); NK cells were stained with mAbs against CD335 purchased from Invitrogen (Carlsbad, CA, USA); eosinophils were stained with mAbs against CCR3 and SigleCF purchased from R&D Systems (Minneapolis, MN, USA) and BD Biosciences (Carlsbad, CA, USA), respectively; DC cells were stained with mAbs against CD11c and MHC II purchased from Invitrogen (Carlsbad, CA, USA) and BD Biosciences (Carlsbad, CA, USA), respectively; macrophages were stained with mAbs against F4/80, CD86 (M1 positive maker), and CD206 (M2 positive maker) purchased from Invitrogen (Carlsbad, CA, USA); B cells were stained with mAbs against CD3e (negative marker) and B220 (positive maker) purchased from Invitrogen (Carlsbad, CA, USA) and BioLegend (San Diego, CA, USA), respectively; T cells were stained with mAbs against CD3e, CD8 (CD8^+^ positive maker), and CD4 (CD4^+^ positive maker) purchased from Invitrogen (Carlsbad, CA, USA). Each sample was analyzed with FACSCalibur and LSR II & LSRFortessa from BD Biosciences, and the data were processed using FlowJo software (Tree Star, Ashland, OR, USA).

##### Toxicological Evaluation

After sacrificing mice, blood samples were collected from the orbital vein. Blood samples were placed in labeled vials containing heparin (5 units mL^−1^) and transferred to 4 °C for analysis and hematological analysis of peripheral circulating blood cells. In the case of serum, the blood was coagulated at RT and then centrifuged at 3000 g at RT for 10 min to separate and then maintain the supernatant. Levels of ALT, AST, Crea, and BUN were measured using the blood biochemical analyzer (BS220, Mindray).

##### Statistical Analysis

Statistical differences between mean values obtained from two sample groups were analyzed using the Student *t*‐test. Differences were considered significant if the *p*‐value was less than or equal to 0.05. Statistical differences across several sample types were analyzed with ANOVA, followed by the Newman−Keuls' multiple comparison test. Asterisks (*, **, and ***) indicate the significance of *p* values less than 0.05, 0.01, and 0.001, respectively.

## Conflict of Interest

The authors declare no conflict of interest.

## Supporting information

Supporting Information

## Data Availability

Research data are not shared.

## References

[1] a) D. M. Smith , J. K. Simon , J. R. Baker Jr. , Nat. Rev. Immunol. 2013, 13, 592. 10.1038/nri3488; 23883969 PMC7097370

[2] Z. J. Deng , M. Liang , M. Monteiro , I. Toth , R. F. Minchin , Nat. Nanotechnol. 2011, 6, 39. 10.1038/nnano.2010.250. 21170037

[3] a) F. Barbero , L. Russo , M. Vitali , J. Piella , I. Salvo , M. L. Borrajo , M. Busquets‐Fite , R. Grandori , N. G. Bastus , E. Casals , V. Puntes , Semin. Immunol. 2017, 34, 52; 29066063 10.1016/j.smim.2017.10.001

[4] a) M. Lundqvist , J. Stigler , G. Elia , I. Lynch , T. Cedervall , K. A. Dawson , Proc. Natl. Acad. Sci. USA 2008, 105, 14265 10.1073/pnas.0805135105; 18809927 PMC2567179

[5] C. Corbo , R. Molinaro , A. Parodi , N. E. Toledano Furman , F. Salvatore , E. Tasciotti , Nanomedicine 2016, 11, 81 10.2217/nnm.15.188. 26653875 PMC4910943

[6] M. Lundqvist , Nat. Nanotechnol. 2013, 8, 701. 24056903 10.1038/nnano.2013.196

[7] S. J. Park , Int. J. Nanomed. 2020, 15, 5783. 10.2147/ijn.s254808. PMC7418457 32821101

[8] a) S. Milani , F. B. Bombelli , A. S. Pitek , K. A. Dawson , J. Radler , ACS Nano 2012, 6, 2532. 10.1021/nn204951s; 22356488

[9] M. Hadjidemetriou , K. Kostarelos , Nat. Nanotechnol. 2017, 12, 288. 28383044 10.1038/nnano.2017.61

[10] a) Y. K. Lee , E. J. Choi , T. J. Webster , S. H. Kim , D. Khang , Int. J. Nanomed. 2015, 10, 97. 10.2147/ijn.s72998; PMC4275058 25565807

[11] a) A. E. Nel , L. Madler , D. Velegol , T. Xia , E. M. Hoek , P. Somasundaran , F. Klaessig , V. Castranova , M. Thompson , Nat. Mater. 2009, 8, 543. 10.1038/nmat2442; 19525947

[12] S. Tenzer , D. Docter , S. Rosfa , A. Wlodarski , J. Kuharev , A. Rekik , S. K. Knauer , C. Bantz , T. Nawroth , C. Bier , J. Sirirattanapan , W. Mann , L. Treuel , R. Zellner , M. Maskos , H. Schild , R. H. Stauber , ACS Nano 2011, 5, 7155. 10.1021/nn201950e. 21866933

[13] a) M. P. Monopoli , D. Walczyk , A. Campbell , G. Elia , I. Lynch , F. B. Bombelli , K. A. Dawson , J. Am. Chem. Soc. 2011, 133, 2525 10.1021/ja107583h; 21288025

[14] a) R. B. Sim , R. Wallis , Nat. Nanotechnol. 2011, 6, 80; 21293484 10.1038/nnano.2011.4

[15] F. Chen , G. Wang , J. I. Griffin , B. Brenneman , N. K. Banda , V. M. Holers , D. S. Backos , L. Wu , S. M. Moghimi , D. Simberg , Nat. Nanotechnol. 2017, 12, 387. 10.1038/nnano.2016.269. 27992410 PMC5617637

[16] M. Neagu , Z. Piperigkou , K. Karamanou , A. B. Engin , A. O. Docea , C. Constantin , C. Negrei , D. Nikitovic , A. Tsatsakis , Arch. Toxicol. 2017, 91, 1031. 27438349 10.1007/s00204-016-1797-5 PMC5316397

[17] a) P. C. Ke , S. Lin , W. J. Parak , T. P. Davis , F. Caruso , ACS Nano 2017, 11, 11773. 10.1021/acsnano.7b08008; 29206030

[18] Y. Lee , S. Fukushima , Y. Bae , S. Hiki , T. Ishii , K. Kataoka , J. Am. Chem. Soc. 2007, 129, 5362. 10.1021/ja071090b. 17408272

[19] E. W. Yu , D. E. Koshland Jr. , Proc. Natl. Acad. Sci. USA 2001, 98, 9517. 10.1073/pnas.161239298. 11504940 PMC55484

[20] X. Zhao , D. Lu , F. Hao , R. Liu , J. Hazard. Mater. 2015, 292, 98. 10.1016/j.jhazmat.2015.03.023. 25797928

[21] N. Sreerama , R. W. Woody , Methods Enzymol. 2004, 383, 318. https://www.ncbi.nlm.nih.gov/pubmed/15063656. 15063656 10.1016/S0076-6879(04)83013-1

[22] a) I. Lynch , K. A. Dawson , Nano Today 2008, 3, 40. 10.1016/s1748-0132(08)70014-8;

[23] M. Raoufi , M. J. Hajipour , S. M. Kamali Shahri , I. Schoen , U. Linn , M. Mahmoudi , Nanoscale 2018, 10, 1228. 10.1039/c7nr06970g. 29292453

[24] M. Mittal , M. R. Siddiqui , K. Tran , S. P. Reddy , A. B. Malik , Antioxid. Redox Signaling 2014, 20, 1126. 10.1089/ars.2012.5149. PMC3929010 23991888

[25] a) V. Bronte , M. J. Pittet , Immunity 2013, 39, 806. 10.1016/j.immuni.2013.10.010; 24238338 PMC3912742

[26] M. Barberis , T. Helikar , P. Verbruggen , Front. Physiol. 2018, 9, 879. 30116196 10.3389/fphys.2018.00879 PMC6083814

[27] N. D. Pennock , J. T. White , E. W. Cross , E. E. Cheney , B. A. Tamburini , R. M. Kedl , Adv. Physiol. Educ. 2013, 37, 273. 10.1152/advan.00066.2013. 24292902 PMC4089090

[28] a) C. C. Fleischer , C. K. Payne , Acc. Chem. Res. 2014, 47, 2651. 10.1021/ar500190q; 25014679 PMC4139184

[29] J. Rosenberg , J. Huang , Curr. Opin. Chem. Eng. 2018, 19, 9. 10.1016/j.coche.2017.11.006. 29623254 PMC5880541

[30] Y. K. Lee , J. Choi , W. Wang , S. Lee , T. H. Nam , W. S. Choi , C. J. Kim , J. K. Lee , S. H. Kim , S. S. Kang , D. Khang , ACS Nano 2013, 7, 8484. 10.1021/nn4041206. 24028581

[31] Z. Liu , S. M. Tabakman , Z. Chen , H. J. Dai , Nat. Protoc. 2009, 4, 1372. 10.1038/nprot.2009.146. 19730421 PMC2853228

[32] E. H. Lee , C. W. Park , Y. J. Jung , J. Ethnopharmacol. 2013, 146, 608. 10.1016/j.jep.2013.01.035. 23384785

