# Supplementary material for: Unfolded Protein Corona Surrounding Nanotubes Influence the Innate and Adaptive Immune System
Source: Adv Sci (Weinh). 2021 Mar 1;8(8):2004979. doi: 10.1002/advs.202004979 (PMC8061349; doi:10.1002/advs.202004979)
Supplement: Supplementary file 1 — Supporting Information [file ADVS-8-2004979-s001.pdf]

© 2021 Wiley-VCH GmbH

ADVANCED SCIENCE
Open Access

Supporting Information

for *Adv. Sci.*, DOI: 10.1002/advs.202004979

Unfolded Protein Corona Surrounding Nanotubes Influence Innate and Adaptiv e Immune System

Jun-Young Park, Sung Jean Park,* Jun Young Park,* Sang-Hyun Kim, Song Kwon, Yun Jae Jung, and Dongwoo Khang*

Supplementary Figures

Unfolded Protein Corona Surrounding Nanotubes Influence Innate and Adaptive Immune System

Jun-Young Park[1,2,*], Sung Jean Park[3,*], Jun Young Park[2,*], Sang-Hyun Kim[4,*], Song Kwon[1], Yun Jae Jung[1,2,5,†] and Dongwoo Khang[1,2,6,†]

[1]*Lee Gil Ya Cancer and Diabetes Institute, Gachon University, Incheon 21999, South Korea*

[2]*Department of Health Sciences and Technology, GAIHST, Gachon University, Incheon 21999, Korea*

[3]*College of Pharmacy and Gachon Institute of Pharmaceutical Sciences, Gachon University, Incheon 21936, South Korea*

[4]*Department of Pharmacology, School of Medicine, Kyungpook National University, Daegu 41944, South Korea*

[5]*Department of Microbiology, School of Medicine, Gachon University, Incheon 21999, South Korea*

[6]*Department of Physiology, School of Medicine, Gachon University, Incheon 21999, South Korea*

[*]These authors contributed equally to this work

[†]Corresponding Authors:

Prof. Dongwoo Khang

Department of Physiology, College of Medicine, Gachon University, Incheon 21999, South Korea, Phone: +82-32-899-6515, E-mail: dkhang@gachon.ac.kr

Prof. Yun Jae Jung

Department of Microbiology, School of Medicine, Gachon University, Incheon 21999, South Korea, Phone: +82-32-899-6113, E-mail: yjjung@gachon.ac.kr

a

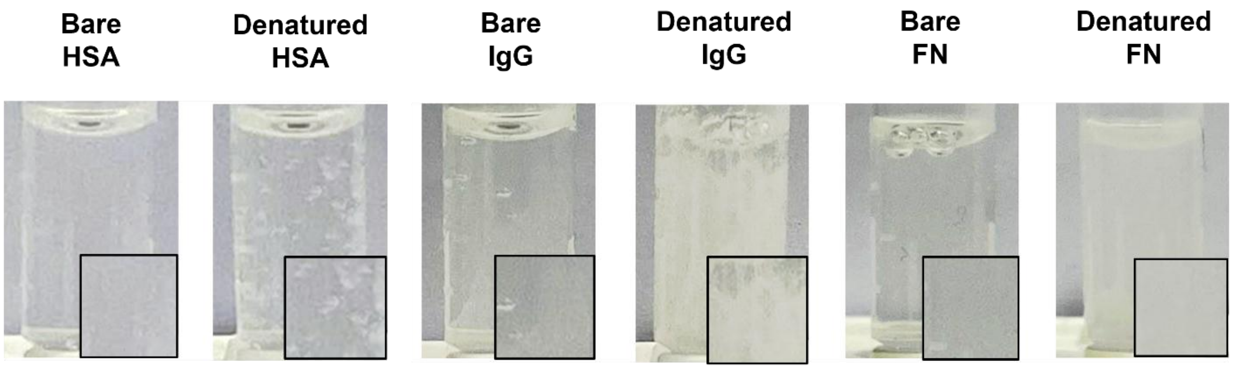

b

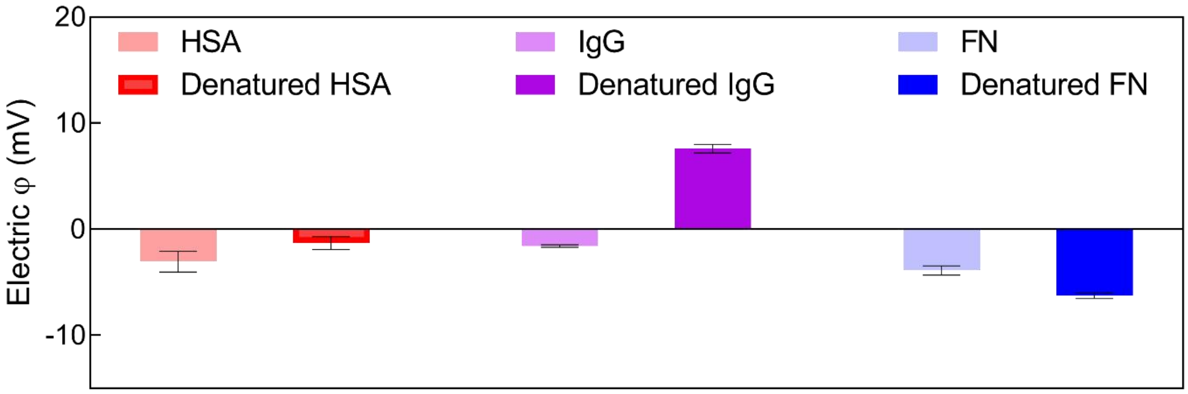

Supplementary Fig. 1.

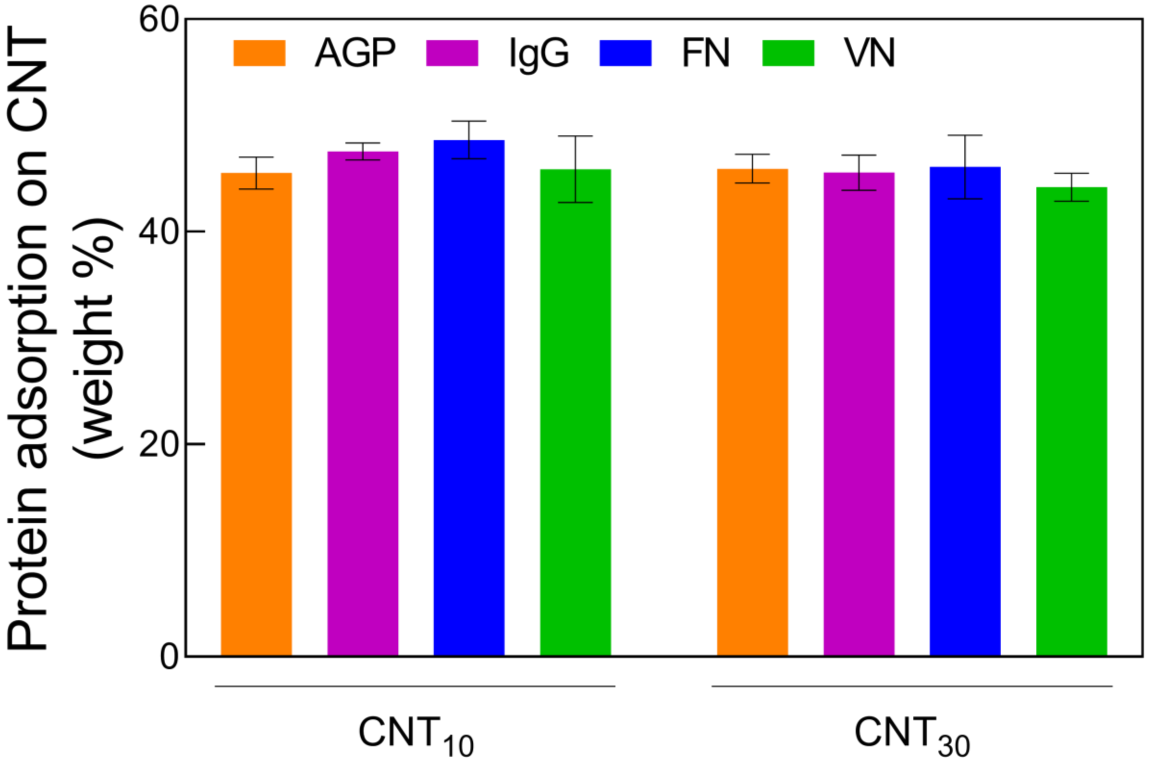

Supplementary Fig. 2.

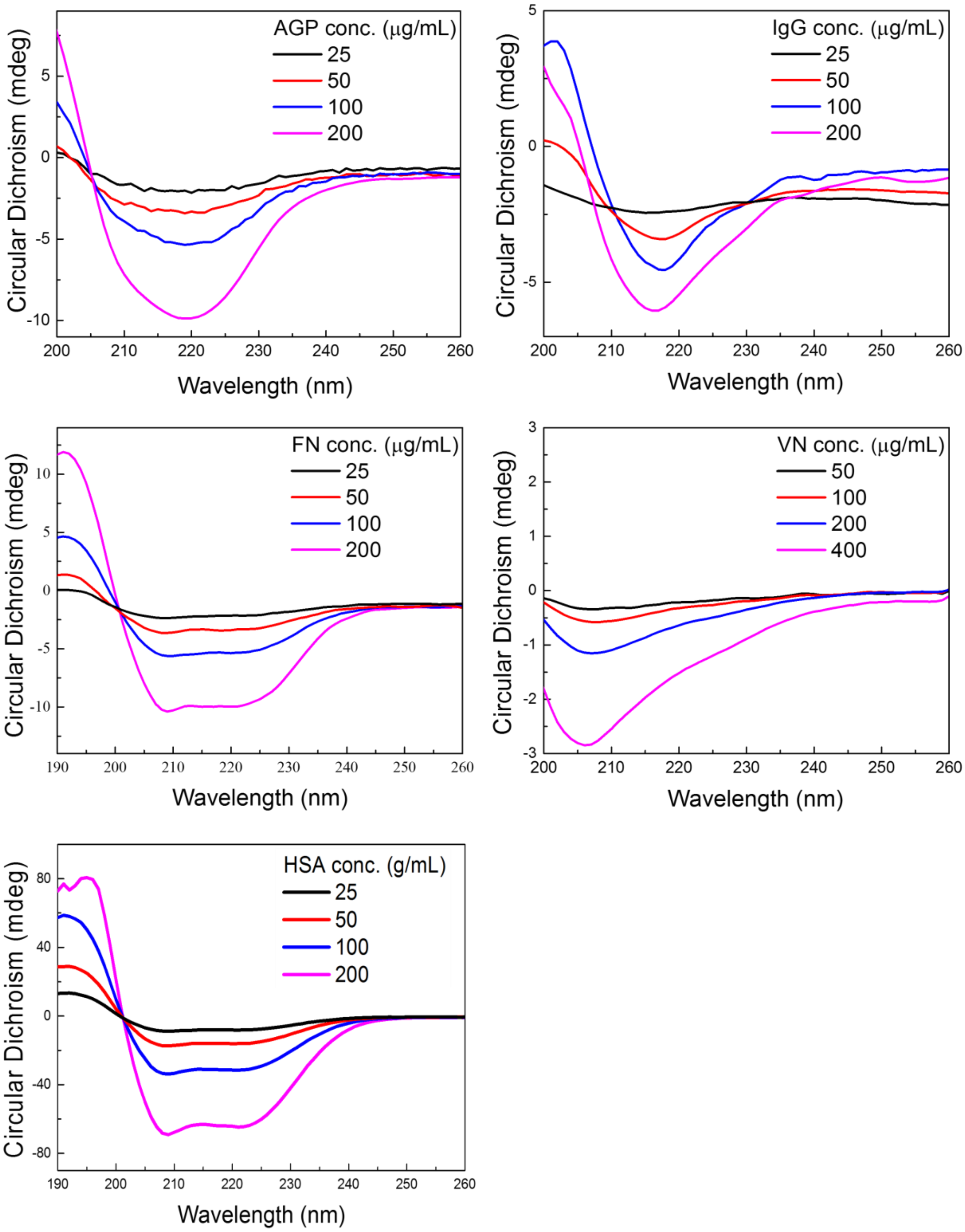

Supplementary Fig. 3.

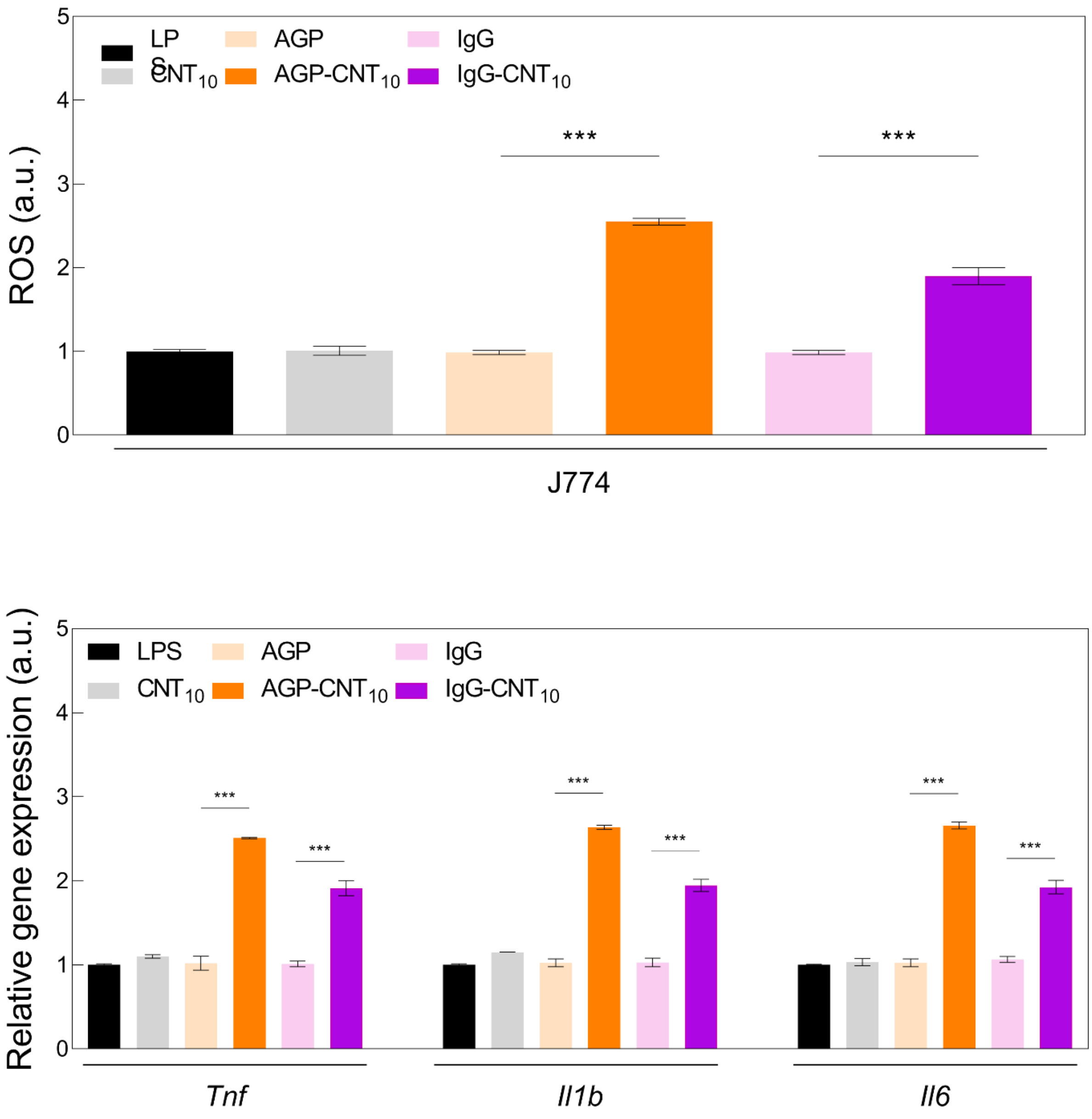

Supplementary Fig. 4.

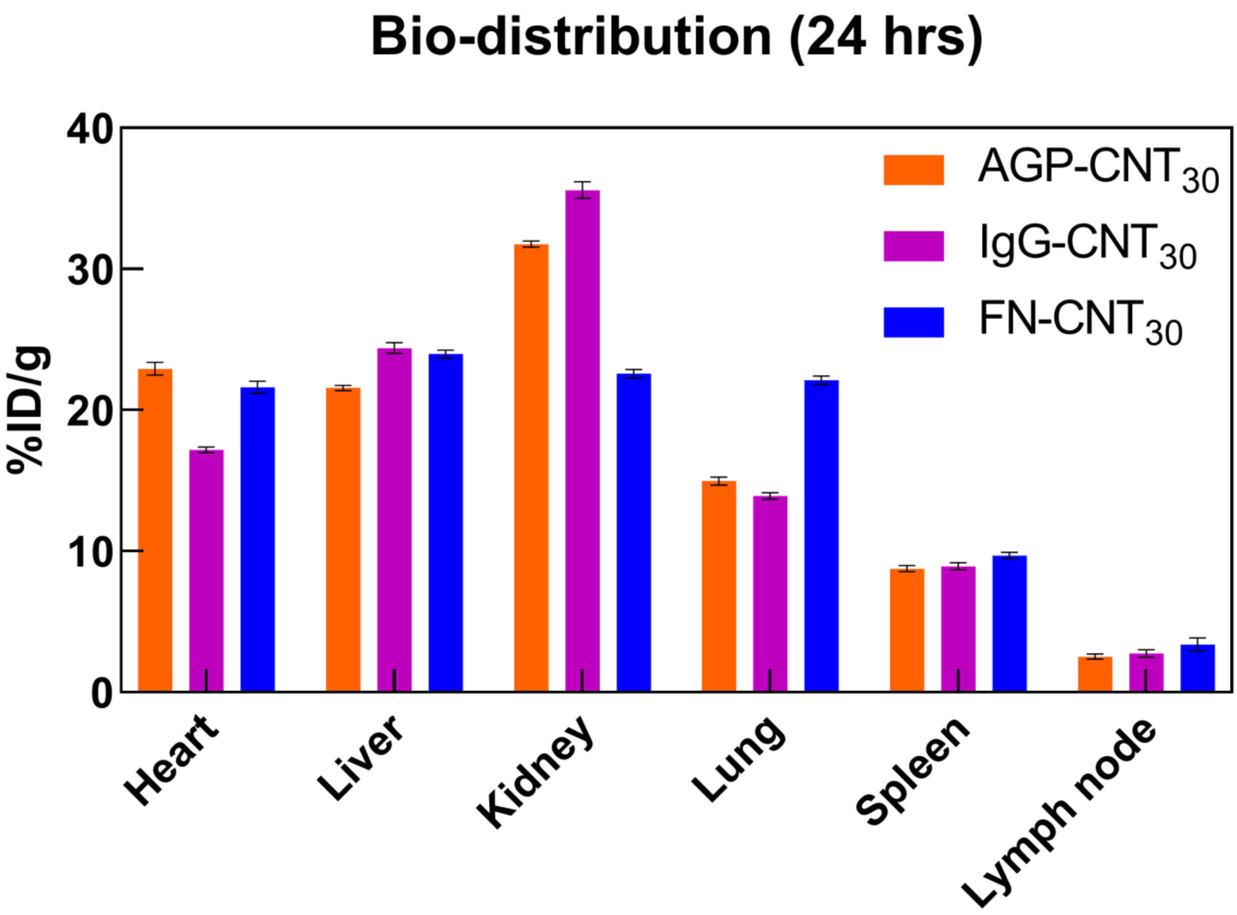

Supplementary Fig. 5.

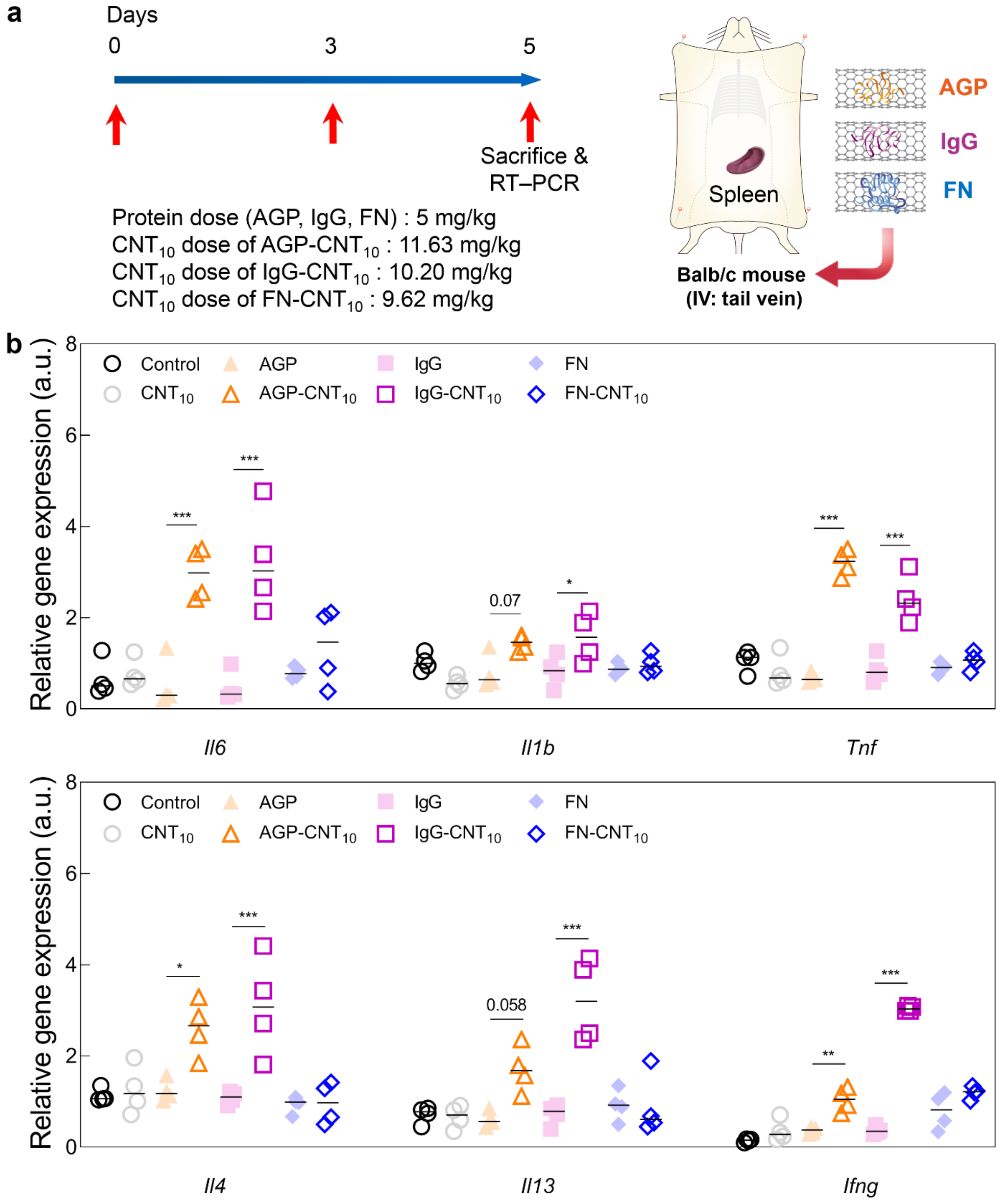

Supplementary Fig. 6.

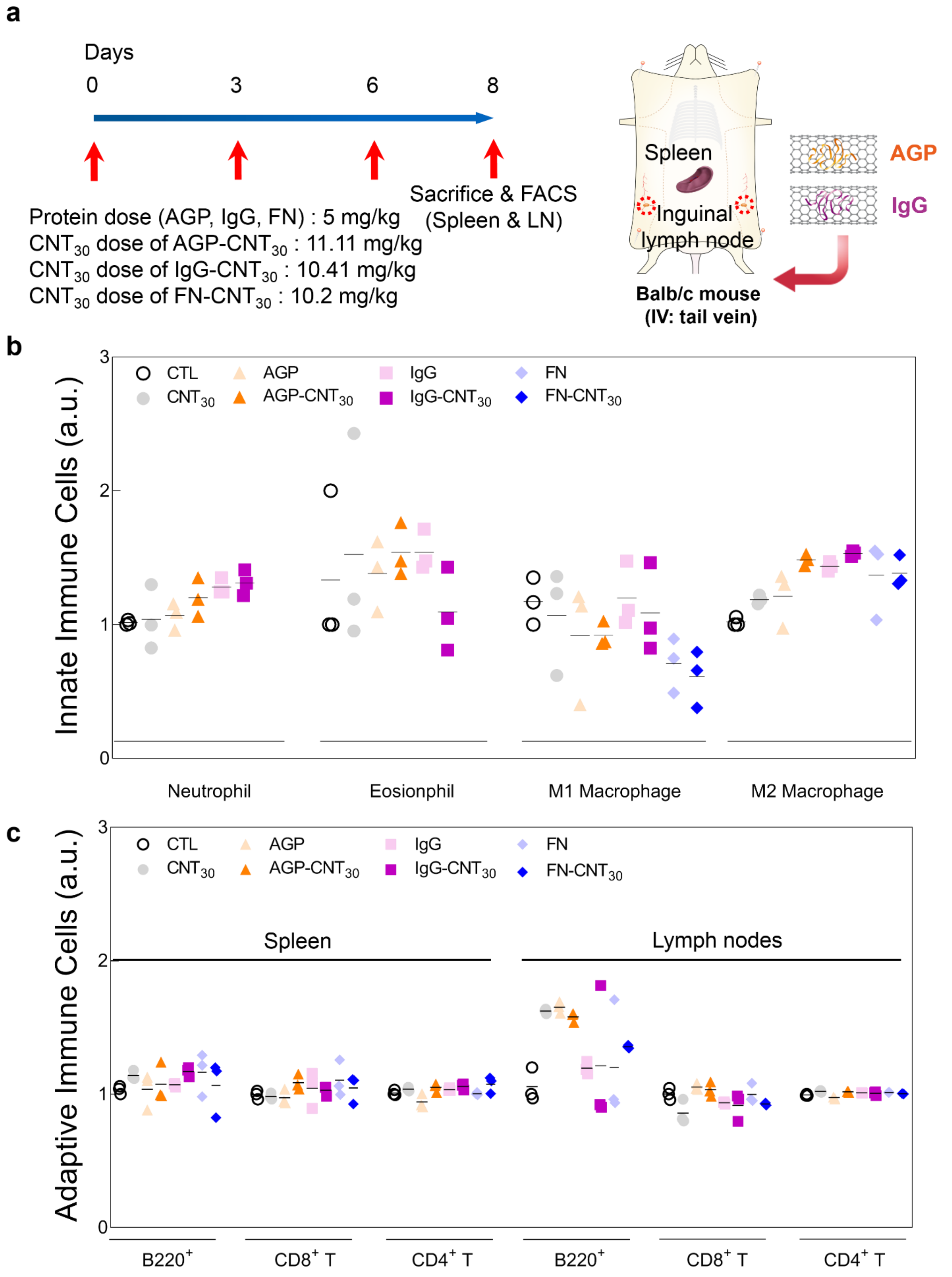

Supplementary Fig. 7.

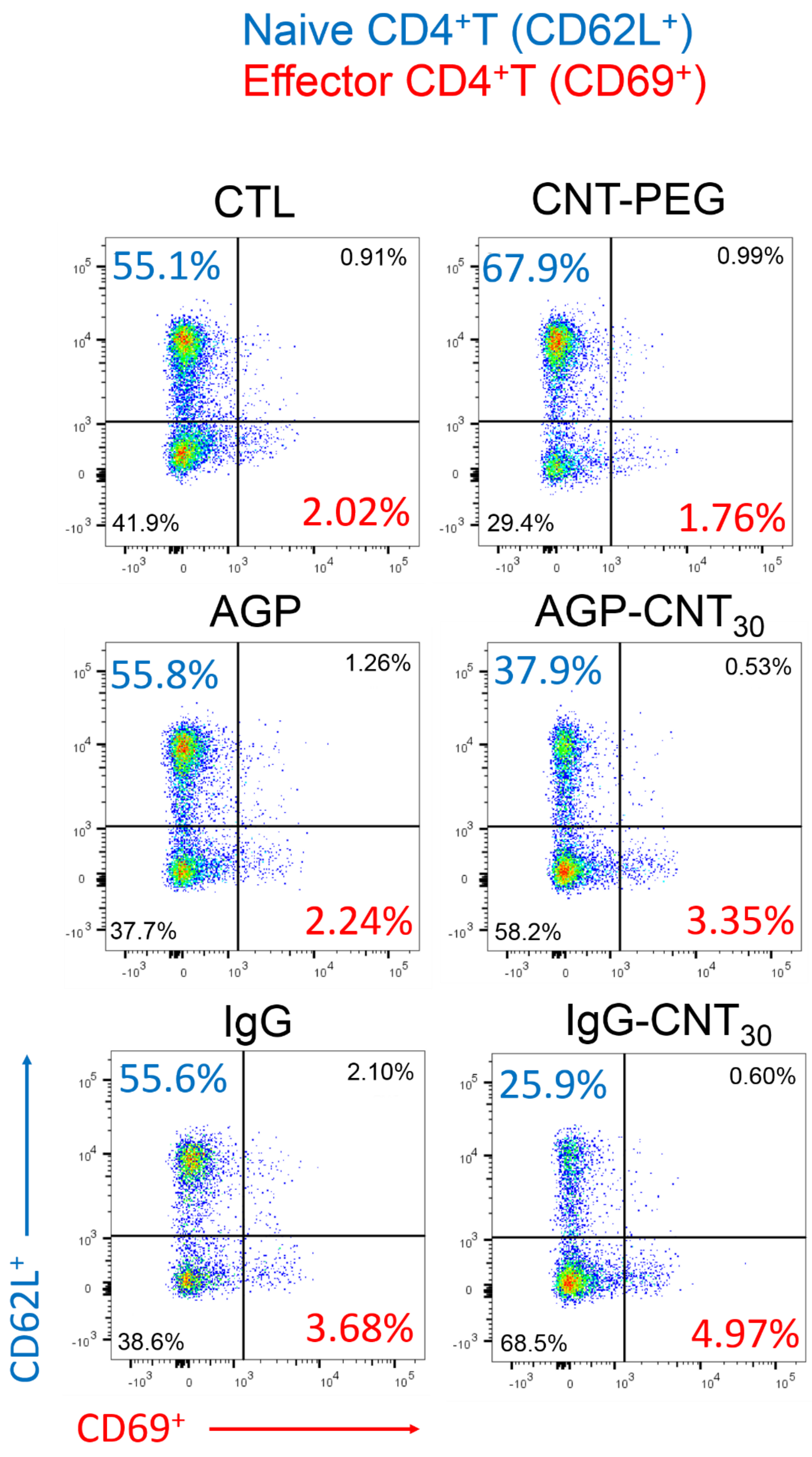

Supplementary Fig. 8.

Target gene	Primer sequence
Il6	Forward : 5'-ATC TTC TCC TGG GGG TAC TG-3'
	Forward : 5'-CTT TTT CTG CAG GAA CTG GA-3'
Tnf	Forward : 5'-CCT ACC AGA CCA AGG TCA AC-3'
	Forward : 5'-AGG GGG TAA TAA AGG GAT TG-3'
Il1b	Forward : 5'-CCA GCT GTA GAG TGG GCT TA-3'
	Forward : 5'-GGA TAT GGA GCA ACA AGT GG-3'
GAPDH	Forward : 5'-GTA TGA CAA CGA ATT TGG CTA CAG-3'
	Forward : 5'-TCT CTC TCT TCC TCT TGT GCT CTT-3'

Supplementary Table. 1.

Mouse primer sequences used for real-time PCR *(in-vitro)*.

Target gene	Primer sequence
Il6	Forward : 5'-TAG TCC TTC CTA CCC CAA TTT CC-3'
	Forward : 5'-TGG TCC TTA GCC ACT CCT TC-3'
Tnf	Forward : 5'-CCT GTA GCC CAC GTC GTA G-3'
	Forward : 5'-GGG AGT AGA CAA GGT ACA ACC C-3'
Il1b	Forward : 5'-GCA ACT GTT CCT GAA CTC AAC T-3'
	Forward : 5'-ATC TTT TGG GGT CCG TCA ACT-3'
Il4	Forward : 5'-CTG TAG GGC TTC CAA GGT GCT TCG-3'
	Forward : 5'-CCA TTT GCA TGA TGC TCT TTA GGC-3'
Il13	Forward : 5'-CCT GGC TCT TGC TTG CCT T-3'
	Forward : 5'-GGT CTT GTG TGA TGT TGC TCA-3'
Infg	Forward : 5'-ATG AAC GCT ACA CAC TGC ATC-3'
	Forward : 5'-CCA TCC TTT TGC CAG TTC CTC-3'
GAPDH	Forward : 5'-CTG GTA TGA CAA TGA ATA CG-3'
	Forward : 5'-GCA GCG AAC TTT ATT GAT GG-3'

Supplementary Table. 2.

Mouse primer sequences used for real-time PCR *(in-vivo)*.
